# Pediatric Anesthesia

**Published:** 2019-01-12

**Authors:** S Tesoro, L Marchesini, E De Robertis

**Affiliations:** Division of Anaesthesia, Analgesia, and Intensive Care, Department of Surgical and Biomedical Sciences - University of Perugia, Italy

Pediatric anesthesia has evolved as a subspecialty because the needs of infants and young children are fundamentally different from those of adults: they differ anatomically, physiologically, pharmacologically, emotionally and socially. Children who undergo anesthesia have special needs and all children have the right to receive the highest attainable standard of health in the right environment. The pediatric patient subjected to anesthesia is burdened by a significant increase in perioperative severe critical events that require unplanned immediate intervention to prevent the occurrence of disability or death. A recent pan-Europe multicentre study^1^ revealed a high rate of severe critical events (5.2% 95%CI 5.0–5.5) in a large peri-operative pediatric cohort with an incidence of respiratory critical events of 3.1%. Cardiovascular instability occurred in 1.9%, with an immediate poor outcome in 5.4% of these cases. The all-cause 30-day in-hospital mortality rate was 10 in 10 000. This was independent of type of anaesthesia. Age (relative risk 0·88, 95% CI 0·86–0·90; p<0·0001), medical history, and physical condition (1·60, 1·40–1·82; p<0·0001) were the major risk factors for a serious critical event. Multivariate analysis revealed evidence for the beneficial effect of years of experience of the most senior anaesthesia team member (0·99, 0·981–0·997; p<0·0048 for respiratory critical events, and 0·98, 0·97–0·99; p=0·0039 for cardiovascular critical events).

These important and recent data invite us to be aware that anesthesia for children should always be delivered by competent and appropriately experienced anesthesiologists and care teams. Children aged less than 3 years, ASA physical status ≥ III (all ages), with underlying congenital and metabolic diseases and/or undergoing major or complex surgery are those at highest risk for perioperative complications and poor outcome. It is recommended that these children should receive care by an anesthesiologist with specific education, training and ongoing experience in pediatric anesthesia. Anesthesiologists with mixed practice can deliver pediatric anesthesia for healthy children aged >3 years simple and routine procedures provided they have the adequate expertise and regular practice in pediatric anesthesia. They should maintain their competence and skills with regular exposure to pediatric lists and keep their knowledge up-to-date. The practice of pediatric anesthesia should be accompanied by continuous professional development (CPD).

How to become a pediatric anesthesiologist? There is still no recognized framework for common and homogeneous pediatric anesthesia training in Europe. In the 90’s FEAPA (Federation of European Association of Pediatric Anesthesia later became ESPA European Society of Pediatric Anesthesia) made recommendations to certify training in pediatric anesthesia which were transposed unevenly in various European countries.^2^ Currently in Europe is required a minimum pediatric anesthesia training for general anesthesia certification and a specific curriculum for specialist pediatric anesthesiologist and periodic revalidation through CPD (Continuous Professional Development). Scientific Societies have developed specific programs aimed at perfecting and implementing some subspecialties through modules of professional development (MPD) that are in-depth concerning topics of interest as revealed by the needs assessment process: an example could be Pediatric Anesthesia.

However still today there are so many legislative differences among European countries that regulate the statutes of postgraduate training and also in the countries where pediatric anesthesia is recognized as a subspeciality (UK, Scandinavian countries and France) the duration of the fellowship, the supervision and the quality of training are different. There are currently only four national programs for maintenance of anesthesia certification, and these are available only in three countries (United states, Canada, UK)^3^. It is expected that all certified anesthesiologists are able to assess an emergency situation, recognize the seriousness and severity of the case, and stabilize the patient before handover to a referral center or specialist pediatric anesthesiologist or intensivist.

In Italy, during postgraduate training we have pediatric anesthesia as part of compulsory professional activity (attività professionalizzante obbligatoria) in the area core specialistic skills, but the duration and the quality of this training is very different among the University Postgraduate School. So SARNePI (Società Anestesia e Rianimazione Neonatale e Pediatrica Italiana) and SIAARTI (Società Italiana di Anestesia Analgesia Rianimazione e Terapia Intensiva) have recently drafted a document in which they define who does what in the field of pediatric anesthesia.^4^ The document defines clinical and organizational recommendations to limit the risk of major complications during elective sedation and anesthesia procedures, both general and regional, in the pediatric age. The purpose of the document is to ensure the best standard of care zeroing the clinical risk throughout the perioperative period. The recommendations defined the number and type of procedures necessary to acquire and maintain the skills for the management of pediatric anesthesia, and the right environment because it’ s very important that all children must be admitted to pediatric wards and must be managed by personnel with specific pediatric experience.

Children should receive pediatric anesthesia care in children’s hospitals or in general/district hospitals with dedicated pediatric areas. The pediatric population is too small to allow maintenance of sufficient skills for every anesthesiologist. Care of children undergoing anesthesia should ideally be regionalized in specialized pediatric settings staffed by pediatric anesthesiologists and pediatric nurses. In particular, the most vulnerable pediatric population, such as neonates and infants, those with co-morbidities and with underlying congenital and metabolic diseases as well as children undergoing major or complex surgery require referral to appropriately resourced multidisciplinary pediatric environments. Anesthesia for elective routine operations in otherwise stable and healthy children can be performed in district hospitals by teams with sufficient expertise in pediatric anesthesia. This requires appropriate staffing, equipment, facilities and support services.

The most of pediatric surgery is performed in a healthy population, has a short duration and a recovery with few complications, so infants and children are well suited for ambulatory surgery. The frequency of pediatric ambulatory surgery has exploded such that 80–90% of pediatric surgery are now performed as ambulatory surgery^5^. The growing awareness that surgical and anesthesiological activities should be understood as risk processes has resulted in the requirement to create a decision-making process that is capable of delivering high quality performance in a restricted hospitalization time, ensuring maximum safety and a reduction of costs.^6^ So ambulatory surgery is the standard for the majority of pediatric surgery in 2019 and today also numerous interventions performed in laparoscopy are suitable in a day surgery regime.^7^ Adenotonsillectomy is the second most common ambulatory surgery in children. Adenotonsillectomy is also one of the interventions in the pediatric population most at risk of developing complications in the perioperative period^8^, especially when the child’s age is less than three years, there are comorbidity and the severity of the obstructive sleep apnea syndrome (OSAS) is high. Therefore the management of an apparently simple and widespread intervention such as the adenotonsillectomy must provide a well-defined clinical organizational pathway performed by staff with expertise in the management of the pediatric populations. Adenotonsillectomy can be considered the absurdity of pediatric anesthesia?

The data we have available requires us centralizing children in the structure of reference with appropriately experienced anesthesiologists and care teams, but can we centralize all healthy children who undergo minor surgery? The answer is NO but the maximum competence of the personnel must be assured by guaranteeing a certain volume of activity. An adequate environment suitable for children with safety standards that must absolutely be present and centralize the most complex cases for pathology, for age and for comorbidity.
